# Rare Case of Tongue Metastasis From Lung Adenocarcinoma, With White Coating Appearance

**DOI:** 10.1002/rcr2.70256

**Published:** 2025-07-04

**Authors:** Toshiki Morimoto, Masahiro Tahara, Azusa Takahashi, Maika Meguro, Yukinori Maeda, Taiga Nagasawa, Hiroaki Degawa, Takanobu Jotatsu, Hiroshi Mukae, Kazuhiro Yatera

**Affiliations:** ^1^ Department of Respiratory Medicine University of Occupational and Environmental Health, Japan Kitakyushu Japan; ^2^ Department of Otorhinolaryngology‐Head and Neck Surgery University of Occupational and Environmental Health, Japan Kitakyushu Japan; ^3^ Department of Respiratory Medicine Graduate School of Biomedical Sciences, Nagasaki University Nagasaki Japan

**Keywords:** lung cancer, tongue metastasis, white coating

## Abstract

Tongue metastasis from other organs is rare, especially from lung cancer. However, in lung cancer cases, the tongue should be considered as a potential site for distant spread.

## Graphical abstract

We present a rare case of tongue metastasis from lung adenocarcinoma. Our patient first presented a very unique macroscopic finding of white coating on the tongue with tongue metastasis from lung cancer. The gross appearance of tongue metastases may vary from ulcerated to polypoid, suggesting that the white coating in our patient was due to ulceration. Tongue metastasis should be considered as a potential site of distant spread in patients with lung cancer.

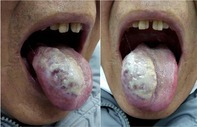

A 78‐year‐old Japanese man with a 58‐pack‐year smoking history presented to our hospital with a rapidly growing tongue tumour. Physical examination revealed a 34 mm tumour with a white coating on the right lateral border of the tongue (Figure 1), and the biopsy obtained from this tumour showed poorly differentiated adenocarcinoma without fungal elements (Figure 2). Chest computed tomography revealed a 45 mm lung tumour in the right lower lobe of the lung and mediastinal lymph node enlargement (Figure 3), and pathological findings of transbronchial biopsy specimens obtained from the right lower lobe tumour were consistent with the results of the tongue biopsy (Figure 4), confirming the final diagnosis of primary lung adenocarcinoma with tongue and bilateral adrenal metastasis. The patient received radiation therapy for the tongue metastasis and was administered carboplatin and nab‐paclitaxel; however, due to rapid progression of the lung cancer, the patient died 1 month after treatment initiation. Metastasis to the tongue from distant primary organs is rare, with a reported incidence of tongue metastases from non‐small cell lung cancer ranging from only 0.2% to 1.6% [1]. There are very few reported cases of primary tumours metastasising to the tongue from lung cancer [2] including the present case. Our patient first presented a very unique macroscopic finding of white coating with tongue metastasis from lung cancer. The gross appearance of tongue metastases may vary from ulcerated to polypoid [2], suggesting that the white coating in our patient might be due to ulceration. Tongue metastasis should be considered as a potential site of distant spread in patients with lung cancer.

**FIGURE 1:**
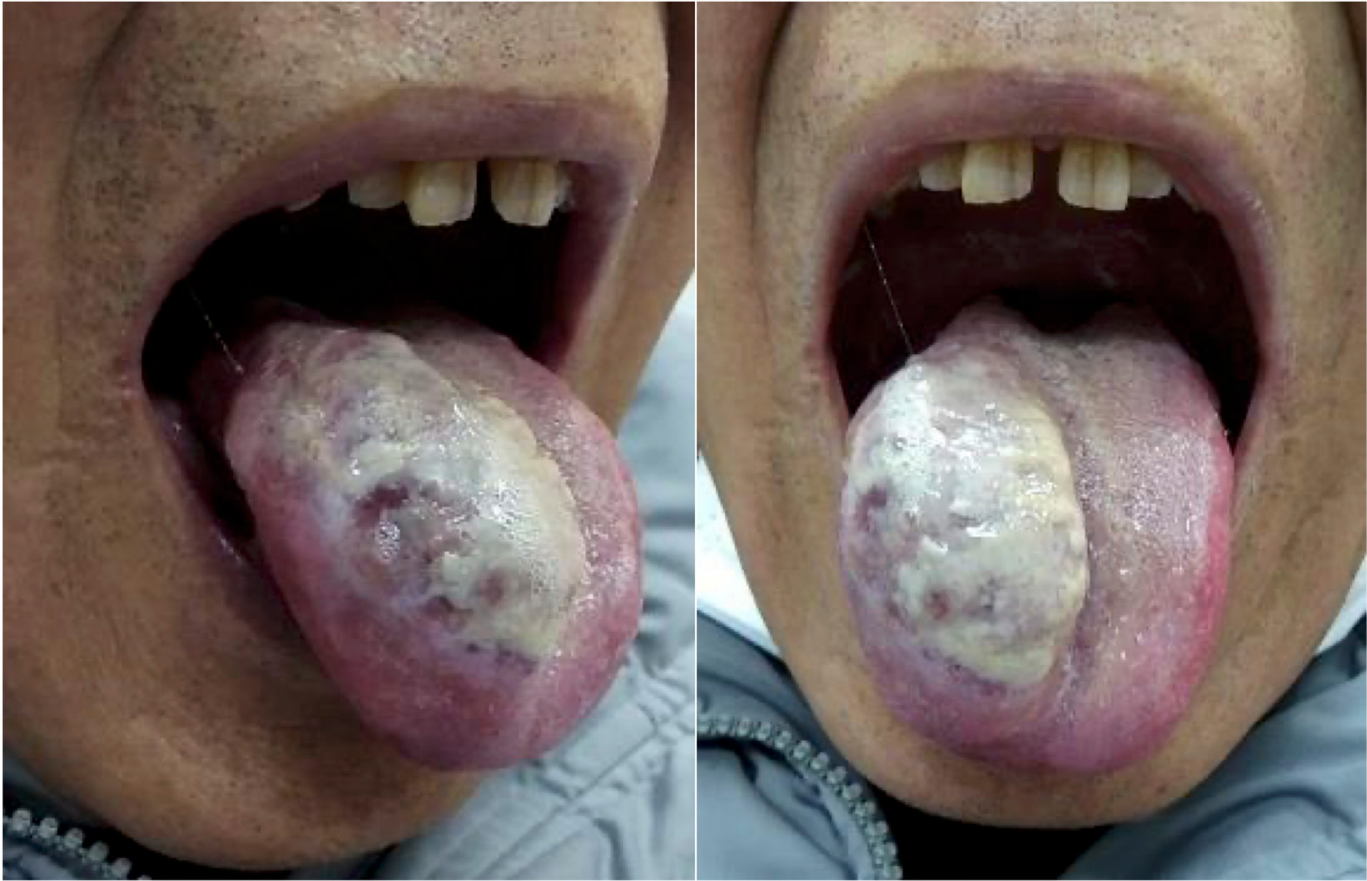
Physical findings of the tongue; there was a white‐coated lesion present on the right lateral aspect of the tongue.

**FIGURE 2:**
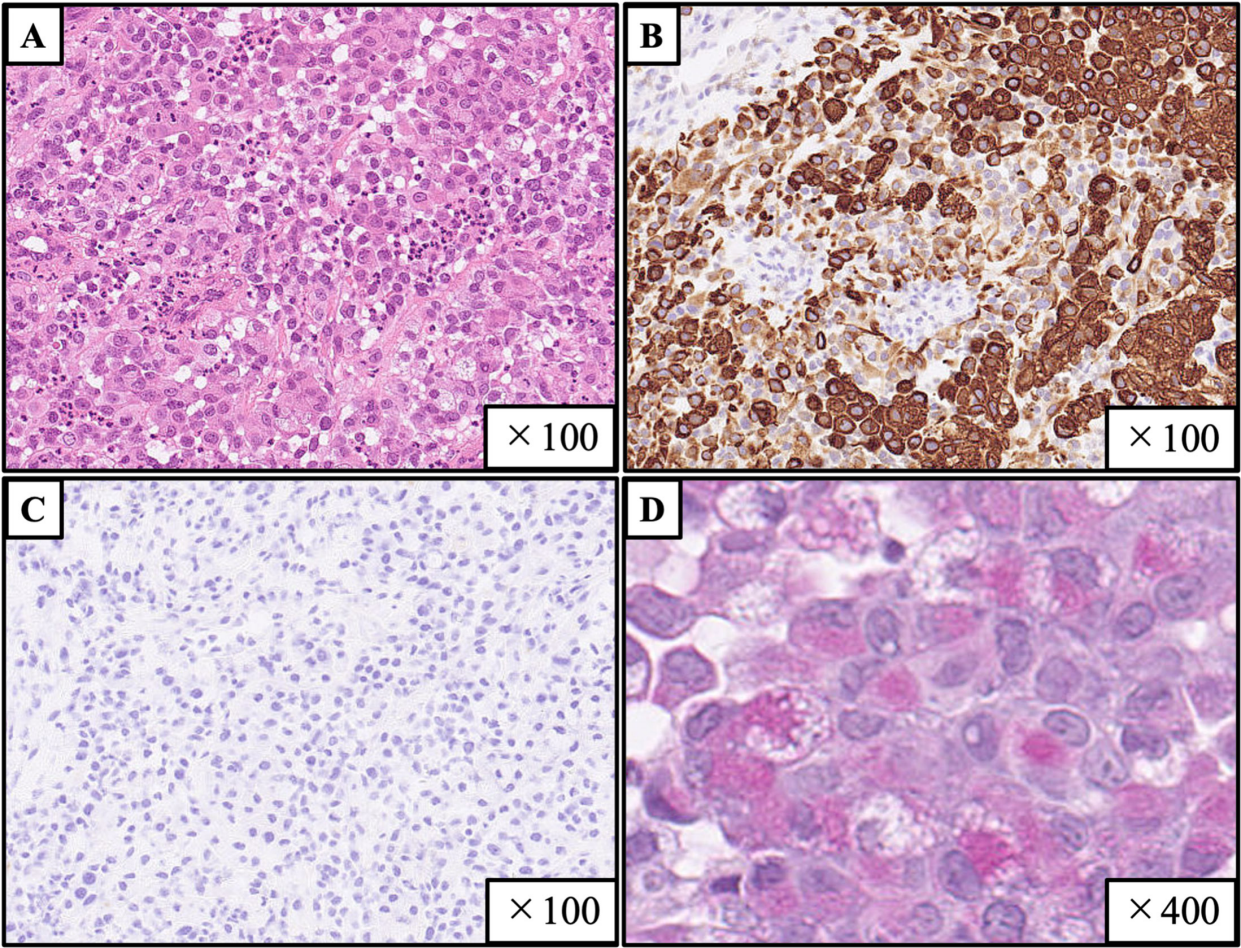
Histopathological findings of the biopsy specimens obtained from tongue tumour. Haematoxylin–eosin staining reveals atypical cells with irregular nuclei of various sizes proliferating within a fibrous stroma. (A) Immunohistochemically, the tumour cells are positive for cytokeratin 7, (B) and negative for cytokeratin 20, (C), and some of these tumour cells are also positive for mucicarmine (intracellular mucin) (D).

**FIGURE 3:**
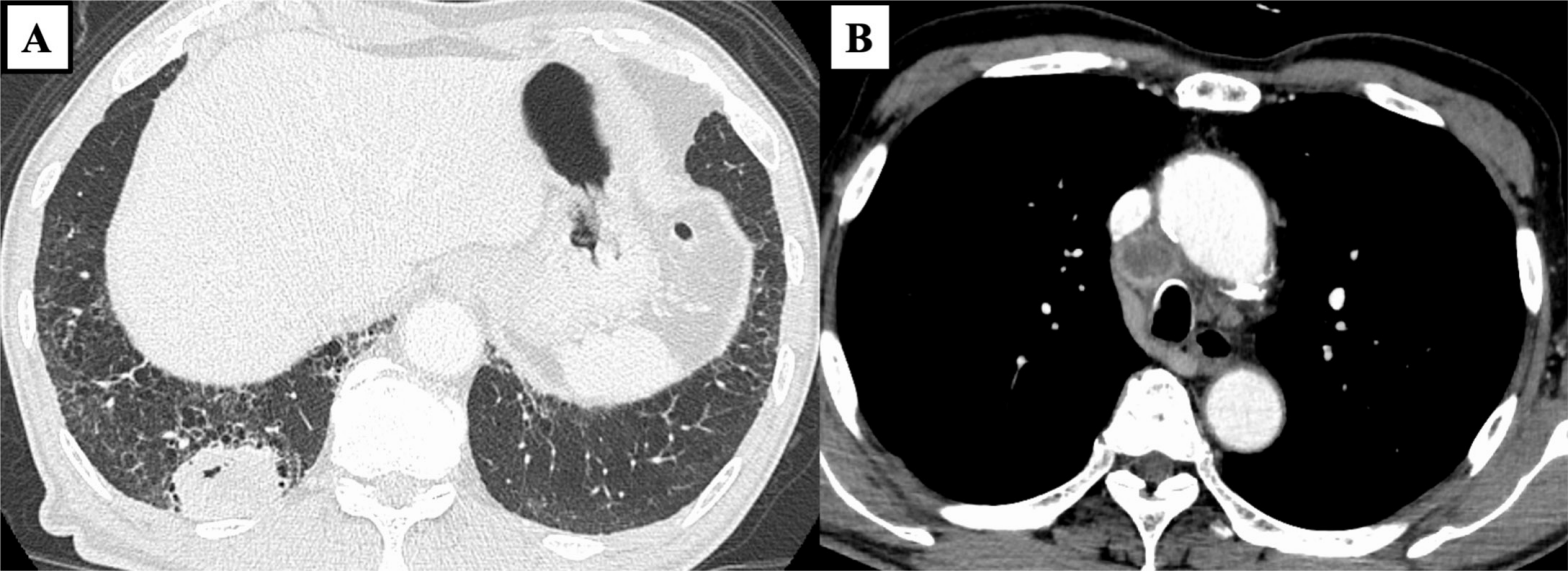
Chest computed tomography upon admission revealed a 45 mm tumour shadow in the right lower lobe of the lung (yellow arrows) and mediastinal lymph node enlargement (green arrow).

**FIGURE 4:**
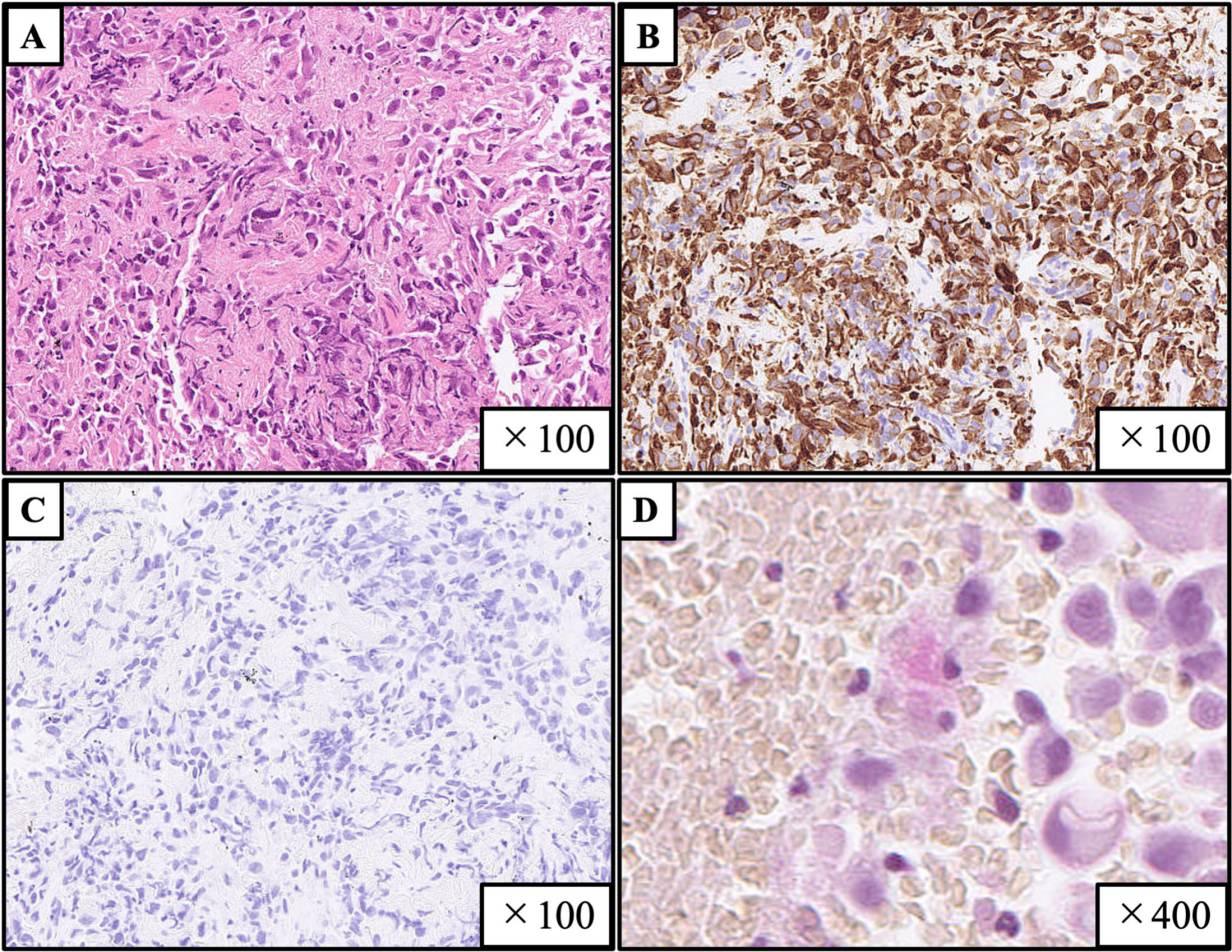
Histopathological findings of the right lung tumour tissue. Almost identical histological findings to the tongue tumour in haematoxylin–eosin staining (A), stainings for cytokeratin 7 (B), cytokeratin 20 (C), and mucicarmine (D).

## Author Contributions

Toshiki Morimoto drafted the manuscript. Azusa Takahashi performed a biopsy of the tongue. Yukinori Maeda performed bronchoscopy and critically revised the manuscript for important intellectual content. Maika Meguro, Taiga Nagasawa, and Hiroaki Degawa critically revised the manuscript for important intellectual content. Masahiro Tahara, Takanobu Jotatsu, Hiroshi Mukae, and Kazuhiro Yatera critically revised the manuscript.

## Ethics Statement

The authors declare that appropriate written informed consent was obtained for the publication of this manuscript and accompanying images.

## Conflicts of Interest

The authors declare no conflicts of interest.

## Data Availability

The data that support the findings of this study are available from the corresponding author upon reasonable request.
